# Supplementary material for: Mapping the evolution of fertility support policies in China: A content and instrumental analysis
Source: PLoS One. 2025 Oct 9;20(10):e0332137. doi: 10.1371/journal.pone.0332137 (PMC12510515; doi:10.1371/journal.pone.0332137)
Supplement: S1 Appendix — (ZIP) [file pone.0332137.s001.zip › S1 Appendix. 226 original policy documents/142-中共中央办公厅、国务院办公厅印发《深圳建设中国特色社会主义先行示范区综合改革试点实施方案(2020-2025年)》(FBM-CLI-16-346747).doc]

中共中央办公厅、国务院办公厅印发《深圳建设中国特色社会主义先行示范区综合改革试点实施方案(2020－2025年)》

**发布部门：** [中共中央办公厅](https://www.pkulaw.com/law/chl?Aggs.IssueDepartment=a03) [国务院办公厅](https://www.pkulaw.com/law/chl?Aggs.IssueDepartment=601) [机构沿革](javascript:void(0))

**发布日期：**2020.10.11

**实施日期：**2020.10.11

**时效性：** [现行有效](https://www.pkulaw.com/law/chl?Aggs.TimelinessDic=01)

**效力级别：** [党内法规制度](https://www.pkulaw.com/law/chl?Aggs.EffectivenessDic=XR12)

**法规类别：** [机关工作综合规定](https://www.pkulaw.com/law/chl?Aggs.Category=00301)

中共中央办公厅、国务院办公厅印发《深圳建设中国特色社会主义先行示范区综合改革试点实施方案（2020－2025年）》

　　近日，中共中央办公厅、国务院办公厅印发了《深圳建设中国特色社会主义先行示范区综合改革试点实施方案（2020－2025年）》，并发出通知，要求各地区各部门结合实际认真贯彻落实。
　　《深圳建设中国特色社会主义先行示范区综合改革试点实施方案（2020－2025年）》主要内容如下。
　　党中央作出兴办经济特区重大战略部署40年来，深圳敢闯敢试、敢为人先、埋头苦干，创造了发展史上的奇迹，成为全国改革开放的一面旗帜。2019年8月，以习近平同志为核心的党中央作出支持深圳建设中国特色社会主义先行示范区的重大决策，一年来，各项工作取得积极进展。以设立经济特区40周年为契机，在中央改革顶层设计和战略部署下，支持深圳实施综合授权改革试点，是新时代推动深圳改革开放再出发的又一重大举措，是建设中国特色社会主义先行示范区的关键一招，也是创新改革方式方法的全新探索，为贯彻落实习近平总书记关于深圳改革发展的重要指示批示精神和《[中共中央、国务院关于支持深圳建设中国特色社会主义先行示范区的意见](https://www.pkulaw.com/chl/0102f7a88cf4b42fbdfb.html)》有关要求，积极稳妥做好综合授权改革试点工作，制定本方案。
　　一、总体要求
　　（一）指导思想。以习近平新时代中国特色社会主义思想为指导，全面贯彻党的十九大和十九届二中、三中、四中全会精神，按照党中央、国务院决策部署，牢牢把握正确改革方向，围绕中国特色社会主义先行示范区的战略定位和战略目标，赋予深圳在重点领域和关键环节改革上更多自主权，支持深圳在更高起点、更高层次、更高目标上推进改革开放，率先完善各方面制度，构建高质量发展体制机制，推进治理体系和治理能力现代化，加快形成全面深化改革、全面扩大开放新格局，推动更高水平深港合作，增强在粤港澳大湾区建设中的核心引擎功能，努力创建社会主义现代化强国的城市范例。
　　（二）工作原则
　　--坚持解放思想、守正创新。坚持和加强党的全面领导，坚持中国特色社会主义道路，坚持以人民为中心的发展思想，在守正的基础上创新，解放思想、实事求是，固根基、扬优势、补短板、强弱项。
　　--坚持市场化、法治化、国际化。充分发挥市场在资源配置中的决定性作用，更好发挥政府作用，推进改革与法治双轮驱动，实施更大范围、更宽领域、更深层次的全面开放。
　　--坚持系统集成、协同高效。突出改革系统性、整体性、协同性，聚焦重点领域和关键环节，推动各方面制度更加衔接配套、成熟定型，实现改革目标集成、政策集成、效果集成。
　　--坚持先行先试、引领示范。坚持问题导向、目标导向、结果导向，给予充分改革探索空间，在遵循[宪法](https://www.pkulaw.com/chl/7c7e81f43957c58bbdfb.html)和法律、行政法规基本原则前提下，允许深圳立足改革创新实践需要，根据授权开展相关试点试验示范。鼓励大胆创新、真抓实干，注重经验总结，及时规范提升，为全国提供示范。
　　--坚持底线思维、稳步实施。提前预设底线情形，在风险总体可控前提下，科学把握时序、节奏和步骤，分期分步、稳妥有序推进改革。
　　（三）主要目标。2020年，在要素市场化配置、营商环境优化、城市空间统筹利用等重要领域推出一批重大改革措施，制定实施首批综合授权事项清单，推动试点开好局、起好步。2022年，各方面制度建设取得重要进展，形成一批可复制可推广的重大制度成果，试点取得阶段性成效。2025年，重要领域和关键环节改革取得标志性成果，基本完成试点改革任务，为全国制度建设作出重要示范。

　　二、完善要素市场化配置体制机制
　　（四）支持在土地管理制度上深化探索。将国务院可以授权的永久基本农田以外的农用地转为建设用地审批事项委托深圳市政府批准。支持在符合国土空间规划要求的前提下，推进二三产业混合用地。支持盘活利用存量工业用地，探索解决规划调整、土地供应、收益分配、历史遗留用地问题。探索利用存量建设用地进行开发建设的市场化机制，完善闲置土地使用权收回机制。深化深汕特别合作区等区域农村土地制度改革。支持依托公共资源交易平台建设自然资源资产交易市场，完善一二级市场联动的土地市场服务监管体系。试点实行土地二级市场预告登记转让制度。
　　（五）完善适应超大城市特点的劳动力流动制度。深化户籍制度改革，调整完善积分落户政策。完善居住证制度，鼓励根据实际扩大公共服务范围、提高服务标准，稳步推进基本公共服务常住人口全覆盖。允许探索适应新技术、新业态、新产业、新模式发展需要的特殊工时管理制度。
　　（六）支持在资本市场建设上先行先试。推进创业板改革并试点注册制，试点创新企业境内发行股票或存托凭证（CDR）。建立新三板挂牌公司转板上市机制。优化私募基金市场准入环境。探索优化创业投资企业市场准入和发展环境。依法依规开展基础设施领域不动产投资信托基金试点。在中国人民银行数字货币研究所深圳下属机构的基础上成立金融科技创新平台。支持开展数字人民币内部封闭试点测试，推动数字人民币的研发应用和国际合作。
　　（七）加快完善技术成果转化相关制度。改革科研项目立项和组织方式，建立主要由市场决定的科技项目遴选、经费分配、成果评价机制。深化科技成果使用权、处置权和收益权改革，在探索赋予科研人员职务科技成果所有权或长期使用权、成果评价、收益分配等方面先行先试。探索政府资助项目科技成果专利权向发明人或设计人、中小企业转让和利益分配机制，健全国有企业科研成果转化利益分配机制。完善技术成果转化公开交易与监管体系。
　　（八）加快培育数据要素市场。率先完善数据产权制度，探索数据产权保护和利用新机制，建立数据隐私保护制度。试点推进政府数据开放共享。支持建设粤港澳大湾区数据平台，研究论证设立数据交易市场或依托现有交易场所开展数据交易。开展数据生产要素统计核算试点。
　　（九）健全要素市场评价贡献机制。率先探索完善生产要素由市场评价贡献、按贡献决定报酬的机制，增加劳动者特别是一线劳动者劳动报酬。充分尊重科研、技术、管理人才，探索充分体现技术、知识、管理、数据等要素价值的实现形式。深入推进区域性国资国企综合改革试验，支持建立和完善符合市场经济规律与企业家成长规律的国有企业领导人员管理机制，探索与企业市场地位和业绩贡献相匹配、与考核结果紧密挂钩、增量业绩决定增量激励的薪酬分配和长效激励约束机制。

　　三、打造市场化法治化国际化营商环境
　　（十）进一步完善公平开放的市场环境。在全国统一的[市场准入负面清单](https://www.pkulaw.com/chl/fb62cc1e71e4ee0bbdfb.html)基础上，制定深圳放宽市场准入特别措施清单，放宽能源、电信、公用事业、交通运输、教育等领域市场准入。进一步放宽前沿技术领域的外商投资准入限制。支持完善公平竞争制度。完善经营邮政通信业务审批机制。试点能源领域许可审批告知承诺制。推进破产制度和机制的综合配套改革，试行破产预重整制度，完善自然人破产制度。
　　（十一）打造保护知识产权标杆城市。开展新型知识产权法律保护试点，完善互联网信息等数字知识产权财产权益保护制度，探索建立健全证据披露、证据妨碍排除和优势证据规则，建立知识产权侵权惩罚性赔偿制度。探索在部分知识产权案件中实行举证责任转移制度。实施知识产权领域以信用为基础的分级分类监管。
　　（十二）完善行政管理体制和经济特区立法。按程序赋予深圳在干部和机构管理、统筹使用各类编制资源等方面更大自主权。探索完善行政争议多元解决机制，健全行政复议与行政诉讼衔接机制。支持深圳扩宽经济特区立法空间，在新兴领域加强立法探索，依法制定经济特区法规规章。

　　四、完善科技创新环境制度
　　（十三）优化创新资源配置方式和管理机制。支持实行非竞争性、竞争性“双轨制”科研经费投入机制。推动完善科研机构管理机制，建立常态化的政企科技创新咨询制度。实施高层次科技人才定向培养机制。
　　（十四）建立具有国际竞争力的引才用才制度。按程序赋予深圳外国高端人才确认函权限，探索优化外国人来华工作许可和工作类居留许可审批流程。支持探索制定外籍“高精尖缺”人才认定标准，为符合条件的外籍人员办理R字签证和提供出入境便利。为符合条件的外籍高层次人才申请永久居留提供便利。支持探索建立高度便利化的境外专业人才执业制度，放宽境外人员（不包括医疗卫生人员）参加各类职业资格考试的限制。

　　五、完善高水平开放型经济体制
　　（十五）加大制度型开放力度。支持以规则衔接深化粤港澳大湾区合作发展。充分发挥中国（广东）自由贸易试验区深圳前海蛇口片区全面深化改革和扩大开放试验田作用，形成更多可复制可推广的制度创新成果。加强对重大疑难涉外商事案件的业务指导。支持完善法治领域跨境协作机制，健全国际法律服务和纠纷解决机制。
　　（十六）扩大金融业、航运业等对外开放。支持符合条件的在深境内企业赴境外上市融资。开展本外币合一跨境资金池业务试点。支持深圳在推进人民币国际化方面先行先试，推动完善外汇管理体制。支持符合条件的外资金融机构在深圳依法发起设立证券公司、基金管理公司。支持符合条件的外资机构在深圳依法合规获取支付业务许可证。推动构建与国际接轨的金融规则体系。探索完善国际船舶登记制度。赋予深圳国际航行船舶保税加油许可权，进一步放开保税燃料油供应市场。

　　六、完善民生服务供给体制
　　（十七）创新医疗服务体系。支持在深圳开展国际前沿药品临床应用。探索完善医疗服务跨境衔接，建立与国际接轨的医学人才培养、医院评审认证标准体系。支持建设全新机制的医学科学院。支持完善重大疫情防控体制机制，健全公共卫生应急管理体系。
　　（十八）探索扩大办学自主权。探索扩大在深高等学校办学自主权。在符合国家相关政策规定前提下，支持深圳引进境外优质教育资源，开展高水平中外合作办学。赋予深圳对企业博士后科研工作站分站的设立和撤销权限。
　　（十九）优化社会保障机制。探索公共服务多元化供给新机制，加快构建以促进健康为导向的创新型医保制度。支持完善普惠婴幼儿照护服务体系。鼓励利用全国一体化政务服务平台实现医保政务服务一体化办理。支持健全公共就业服务和终身职业技能培训制度。
　　（二十）完善文化体育运营管理体制。支持深化文艺院团改革，完善院团管理体制、运行机制和利益分配制度。按程序赋予省级电视剧审查等管理权限。支持建设适用国际通用规则的文化艺术品（非文物）拍卖中心。支持开展体育消费城市试点，推进体育产业创新试验，创新促进体育赛事发展的服务管理机制和安保制度。

　　七、完善生态环境和城市空间治理体制
　　（二十一）健全生态建设和环境保护制度。支持完善生态保护红线、环境质量底线、资源利用上线和生态环境准入清单等“三线一单”生态环境分区管控体系，开展重要生态空间自然资源确权登记，扩大生态系统服务价值核算范围。支持完善本地清洁能源供应机制，建设能源产业创新中心、创新联合体等平台机构。支持完善产品环保强制性地方标准，建立绿色产业认定规则体系，完善气候投融资机制。推动完善陆海统筹的海洋生态环境保护修复机制，实行环境污染强制责任保险制度，探索建立入海排污口分类管理制度。加快建设国家可持续发展议程创新示范区。
　　（二十二）提升城市空间统筹管理水平。按照相关法律法规，支持推动在建设用地地上、地表和地下分别设立使用权，探索按照海域的水面、水体、海床、底土分别设立使用权，促进空间合理开发利用。开展深化自然生态空间用途管制试点。按程序赋予深圳占用林地省级审核权限。探索优化用地用林用海“统一收文、统一办理、统一发文”审批机制，推动自然资源使用审批全链条融合。开展航空资源结构化改革试点。完善无人机飞行管理制度。

　　八、强化保障措施
　　（二十三）全面加强党的领导。坚持和加强党对深圳建设中国特色社会主义先行示范区综合改革试点的领导，增强“四个意识”、坚定“四个自信”、做到“两个维护”，把党的建设始终贯穿综合改革试点全过程，严密党的组织体系，为深圳深化改革开放提供坚强保障。
　　（二十四）创新工作机制。支持深圳结合实际率先开展相关试点试验示范，实施重大改革举措。国家发展改革委会同有关方面分批次研究制定授权事项清单，按照批量授权方式，按程序报批后推进实施。有关方面要按照本方案要求和经批准的事项清单，依法依规赋予深圳相关管理权限。建立健全重大风险识别及系统性风险防范制度和政策体系。实行包容审慎的改革风险分类分级管控机制，根据风险程度，分别采取调整、暂缓或终止等处置措施，不断优化综合改革试点实施路径。国家发展改革委会同有关部门及时跟进综合改革试点进展，加强统筹协调和指导评估，对达到预期效果的抓紧总结推广，对新情况新问题及时分析评估，重要情况及时向党中央、国务院报告。
　　（二十五）落实地方责任。广东省要积极为深圳开展综合改革试点创造条件，加大行政审批、科技创新、规划管理、综合监管、涉外机构和组织管理等方面放权力度，依法依规赋予深圳更多省级经济社会管理权限。深圳要切实担负起试点主体责任，增强使命感和责任感，认真做好具体实施工作，确保各项改革任务扎实有序推进。要加强与粤港澳大湾区其他城市协调合作，建立区域互动、优势互补的改革联动机制，实现协同对接，充分发挥制度整体效能。
　　（二十六）强化法治保障。建立健全与综合改革试点相配套的法律法规、政策调整机制。本方案提出的各项改革政策举措，凡涉及调整现行法律或行政法规的，经全国人大常委会或国务院授权后实施。有关方面要加强指导和服务，统筹综合改革试点涉及的法律法规事项，做好与相关法律法规立改废释的衔接。
　　（二十七）营造改革氛围。弘扬特区精神，继续大胆地闯、大胆地试。健全改革的正向激励机制，注重在改革一线考察识别干部，大胆提拔使用敢于改革、善于改革的干部，充分调动和激发广大干部参与改革的积极性、主动性和创造性。全面落实“三个区分开来”，建立健全容错纠错机制，宽容干部在改革创新中的失误错误，对干部的失误错误进行综合分析，该容的大胆容，不该容的坚决不容。及时宣传深圳推进改革的新进展新成效，为综合改革试点营造良好舆论氛围。

　　（新华社北京2020年10月11日电）

© 北大法宝：（[www.pkulaw.com](https://www.pkulaw.com/)) 专业提供法律信息、法学知识和法律软件领域各类解决方案。北大法宝为您提供丰富的参考资料，正式引用法规条文时请与标准文本核对。
欢迎查看所有[产品和服务](https://www.pkulaw.com/helps/2.html)。法宝快讯：[如何快速找到您需要的检索结果？](https://www.pkulaw.com/helps/70.html)[法宝](https://www.pkulaw.com/helps/69.html)V6有何新特色？

扫描二维码阅读原文

**原文链接：**<https://www.pkulaw.com/chl/27aaeb8edc6dfa58bdfb.html>
